# Chitosan nanoparticles functionalized with β-cyclodextrin: a promising carrier for botanical pesticides

**DOI:** 10.1038/s41598-018-20602-y

**Published:** 2018-02-01

**Authors:** Estefânia V. R. Campos, Patrícia L. F. Proença, Jhones L. Oliveira, Cirano C. Melville, Jaqueline F. Della Vechia, Daniel J. de Andrade, Leonardo F. Fraceto

**Affiliations:** 1 Department of Environmental Engineering, São Paulo State University (UNESP), Sorocaba, SP Brazil; 2 Department of Biochemistry and Tissue Biology, State University of Campinas, Campinas, SP Brazil; 3 São Paulo State University (UNESP), College of Agricultural and Veterinary Sciences, Jaboticabal, SP Brazil

## Abstract

Carvacrol and linalool are natural compounds extracted from plants and are known for their insecticidal and repellent activities, respectively. However, their low aqueous solubility, high photosensitivity, and high volatility restrict their application in the control of agricultural pests. The encapsulation of volatile compounds can be an effective way of overcoming such problems. Inclusion complexes between beta-cyclodextrin (β-CD) and carvacrol (CVC) or linalool (LNL) were investigated. Inclusion complexes were prepared by the kneading method. Both complexes presented 1:1 host:guest stoichiometry and the highest affinity constants were observed at 20 °C for both molecules. The nanoparticles containing carvacrol and linalool had mean diameters of 175.2 and 245.8 nm, respectively and high encapsulation efficiencies (<90%) were achieved for both compounds. Biological assays with mites (Tetranychus urticae) showed that the nanoparticles possessed repellency, acaricidal, and oviposition activities against this organism. Nanoencapsulated carvacrol and linalool were significantly more effective in terms of acaricidal and oviposition activities, while the unencapsulated compounds showed better repellency activity. The nanoformulations prepared in this study are good candidates for the sustainable and effective use of botanical compounds in agriculture, contributing to the reduction of environmental contamination, as well as promoting the effective control of pests in agriculture.

## Introduction

Invertebrates, weeds, and pathogens, collectively termed pests, have always caused significant losses in agricultural production worldwide. On a global scale, weeds are the greatest cause of agricultural losses, while insects are also responsible for substantial decreases in production^1–4^. The advent of the green revolution led to the discovery and intensification of the use of chemical products for the control of pests and consequently increased agricultural production. However, the inappropriate use of pesticides has led to problems including residues in soil and food, contamination of hydric resources, rapid development of resistant pests, reemergence of pests, and toxicity towards nontarget organisms^3,5–7^. The increasing popularity of organic agriculture, together with the need to overcome problems associated with the emergence of resistant pests, has led to interest in natural pesticides, especially botanical insecticides and repellents, due to their easier degradation and lower toxicity in the environment. Most of these substances are site-specific and present low toxicity towards mammals and nontarget species^6–9^.

Carvacrol (2-methyl-5-[1-methyl-ethyl]phenol) (CVC) is a phenolic monoterpene found in the essential oils of aromatic plants of the genera *Origanum*, *Thymus*, and *Thymbra*, among others. This compound is generally considered safe and is approved by the Food and Drug Administration for use in foods^10^. Many studies have shown that CVC possesses antimicrobial, insecticidal, and antitumor activities, among other properties, as a result of which it finds uses in agriculture, medicine, and the cosmetics and food industries^11^.

Linalool (3,7-dimethyl-1,6-octadien-3-ol), an alcoholic monoterpene found in the essential oils of the genera *Anibia*, *Ocimum*, and *Cinnamomum*, is known to have antifungal^12^ and antimicrobial^12,13^ activities, and is also widely used as an insect repellent^14,15^.

However, important limitations on the use of these compounds in agriculture are: (i) their low aqueous solubility, resulting in reduced contact with pathogens^16,17^, and (ii) their susceptibility to degradation due to exposure to ultraviolet radiation, high temperatures, and oxidation by atmospheric oxygen^18^.

One way to overcome these limitations is by the formation of inclusion complexes with cyclodextrins^19^ and/or encapsulation in nanoparticles^20,21^, resulting in reduced losses by volatilization, photodegradation, and oxidation, as well as increased apparent solubility of the compounds.

Beta-cyclodextrin (β-CD) is a cyclic polysaccharide composed of seven units of glucose (α-D-glucopyranose) linked by α-(1,4) type bonds, which presents a hydrophilic external surface and a hydrophobic internal cavity. This structure enables cyclodextrins to form full inclusion complexes with small molecules or partial inclusion complexes with macromolecules, hence improving the physicochemical and biological properties of the complexed compound^19,22,23^.

Chitosan (CS) is a linear chain polyaminosaccharide obtained from the alkaline deacetylation of chitin. This cationic polymer is composed of D-glucosamine residues linked by β(1→4) glycosidic bonds. Chitosan possesses several valuable structural and functional properties, including biocompatibility, biodegradability, low toxicity, and good miscibility with other polymers, and also has a highly chemically reactive structure, due to the presence of primary amines^24,25^.

A factor that limits the use of chitosan in sustained release applications is its hydrophilicity, resulting in a poor capacity to carry hydrophobic molecules. Efforts have been made to improve the ability of chitosan to carry hydrophobic substances^26,27^, notably by means of functionalization of the chitosan structure with cyclodextrins^28^. In some studies, the advantages of both carriers have been combined in order to develop more efficient carrier systems, such as those providing improved mucoadhesive properties for effective release of drugs for biomedical purposes^29,30^, enhanced protein delivery^23^, sustained release of insecticides^31^, and removal of environmental contaminants^28,32^.

The aim of this work was to prepare and characterize inclusion complexes of carvacrol and linalool with β-CD. In addition, a hybrid polymer was prepared and characterized, and evaluation was made of its ability to form nanoparticles for the encapsulation of carvacrol and linalool. The physicochemical characteristics of the nanoparticles were evaluated in terms of mean diameter, polydispersity index, zeta potential, and encapsulation efficiency. In addition, their biological activities were investigated using a mite (*Tetranychus urticae*) as a model insect. There have been no previous studies concerning the development of chitosan nanoparticles functionalized with cyclodextrin for the encapsulation of carvacrol and linalool, with a view to their use as release systems for these active agents.

## Materials and Methods

### Materials

Carvacrol (98%), linalool (97%), β-cyclodextrin (MW: 1134.98 g/mol), chitosan glycol (≥60% titration), low molecular weight chitosan, N-(3-dimethylaminopropyl)-N′-ethylcarbodiimide (EDC), N-hydroxysuccinamide (NHS), and Tween 80 were purchased from Sigma. The solvent employed for the chromatographic analyses was HPLC grade acetonitrile (JT Baker, Phillipsburg, New Jersey).

### Preparation of the inclusion complexes

The inclusion complexes of carvacrol and linalool with the β-CD were prepared at a 1:1 molar ratio (guest:host), using the kneading method described by Santos *et al*.^33^. A 1 g mass of β-CD was homogenized in a mortar together with 0.132 g of CVC or 0.135 g of LNL, followed by the addition of ethyl alcohol (700 μL) and manually mixing for 45 min. The resulting solid was dried in a desiccator and then stored in a hermetically sealed flask, protected from light. The encapsulation efficiencies (EEs) of the oils in the inclusion complex were determined by the method described by Marreto *et al*.^34^. The quantities of CVC and LNL present in the inclusion complexes were determined using spectrophotometric measurements at 275 and 210 nm for CVC and LNL, respectively. Firstly, the total concentrations of active agents associated with the complexes (present in the cavities and adsorbed on the surfaces) were determined by extraction with acetonitrile:water (95:5, v/v) for 48 h. This was followed by determination of the amount of active agent adsorbed on the surface. The EE was determined as the difference between the initial concentration added and the concentration adsorbed on the surface and entrapped within the cyclodextrin cavity. Quantification employed the following analytical curves: CVC = 0.0112 × + 0.0208 (r^2^ = 0.99386); LNL = 0.0137 × + 0.0496 (r^2^ = 0.99893).

#### Solubility isotherms

Phase solubility assays were performed according to the method described by Higuchi and Connors^35^. Excess CVC or LNL was added to a water:ethanol (95:5, v/v) mixture, followed by addition of increasing amounts of β-CD (0 to 10 mmol/L). The solutions were agitated for 48 h at 200 rpm, after which they were centrifuged and the supernatant was filtered through a 0.22 μm membrane. The concentrations of CVC and LNL were determined spectrophotometrically using wavelengths of 275 and 210 nm, respectively. The experiments were performed in triplicate at different temperatures (20, 25, 30, and 35 °C). The association constant was calculated using Equation 1:

1 $${K}_{c}=\frac{slope}{{S}_{0}.(1-slope)}$$

where *S*_0_ is to the concentration of the bioactive substance in the absence of β-cyclodextrin.

#### Thermodynamic parameters

The solubility isotherm data were used to calculate the thermodynamic properties of the inclusion complexes. The parameters ΔH° and ΔS° are the standard enthalpy and entropy for transfer of carvacrol or linalool from the dispersing medium into the cyclodextrin cavity, respectively, and can be calculated using the van’t Hoff thermodynamic relation (Equation 2).

2 $$\mathrm{ln}\,k=-\frac{{\rm{\Delta }}H\,}{RT\,}+\frac{{\rm{\Delta }}S}{R}$$

where *T* is the temperature (in Kelvin), *R* is the gas constant, and *k* is the affinity constant. For a linear van’t Hoff relation (ln *k* vs. 1/*T*), the slope and the intercept represent ΔH°/*R* and ΔS°/*R*, respectively^36^.

### Preparation of the functionalized chitosan (CSgCD)

Functionalization of the chitosan glycol skeleton by the β-CD was performed according to the methodology described by Tan *et al*.^37^. Firstly, 70 mg of β-CD was dissolved in 70 mL of phosphate buffered saline (PBS) at pH 7, followed by the addition of 125 μL volumes of the EDC and NHS crosslinking agents at concentrations of 0.1 and 0.4 mol L^−1^, respectively. After 6 h of reaction between the β-CD and the crosslinking agents, at ambient temperature, 10.25 mg of chitosan glycol was added and the reaction was continued for a further 18 h. After 24 h of reaction, the final solution was dialyzed against PBS buffer (pH 7) for 24 h, followed by drying of the product in an oven at 35 °C for 3 days.

### Characterization of the inclusion complex and the functionalized chitosan

#### Infrared spectroscopy (FTIR)

Infrared spectra were obtained for the β-CD, chitosan, carboxymethyl chitosan, chitosan glycol, and the functionalized polymer, using a Varian 660 spectrometer equipped with an attenuated total reflectance accessory (GladiATR, Pike Technologies) with a diamond crystal (2.2 × 3.0 mm). The instrument was operated in transmittance mode, in the frequency range from 4000 to 400 cm^−1^, using an incidence angle of 45°, 32 accumulations, and resolution of 8 cm^−1^.

#### X-ray diffraction (XRD)

XRD analyses were performed in transmission mode, using a PANalytical X’Pert^3^ diffractometer. X-ray diffraction patterns were recorded using CuKα radiation, 40 kV voltage, 20 mA current, 0.02° step resolution, and 5 s per step, in the 2θ ranges from 1° to 60° for the CD and the CSgCD^38^.

#### Thermal analyses

Thermal analyses were performed using a TA Instruments DSC Q20 calorimeter equipped with a cooling system. Calibration was achieved using the element indium. The sample (5 mg) was placed in an aluminum sample holder and the thermal profile was obtained between 5 and 300 °C, with heating at a rate of 10 °C/min under a flow of nitrogen (50 mL/min). An empty sample holder was used as a reference. Thermogravimetric analyses were performed using a Shimadzu DTG-60H instrument, with heating between 30 and 300 °C, at a rate of 10 °C/min, under a nitrogen atmosphere.

#### Characterization by nuclear magnetic resonance (NMR)

The ^1^H NMR technique was used to study the formation of the inclusion complexes between the cyclodextrin and the active agents (carvacrol and linalool), as well as the functionalization of the chitosan skeleton with cyclodextrin^39^. Spectra for the samples in water were collected at 25 °C, using a Varian Inova 500 MHz spectrometer equipped with a triple resonance probe. The solvent peak at 4.7 ppm was used as an internal reference. All the spectra were collected using 5 mm tubes, averaging 64 scans and with digital resolution of 0.30 Hz. The residual water signal was suppressed by pre-saturation at low irradiation power. For the inclusion complexes, ROESY spectra were collected using a mixing time of 500 ms, a window of 4000 × 4000 Hz, and acquisition of between 128 and 256 transients with 2 k data points along the t_s_ axis^18^.

### Preparation of functionalized chitosan nanoparticles (CSgCD)

Functionalized chitosan nanoparticles were produced using sodium tripolyphosphate (TPP) as the crosslinking agent, according to the procedure described by Keawchaoon and Yoksan^40^, with some modifications. Chitosan (1.5%, w/v) was dissolved overnight in an aqueous solution of acetic acid (0.5%, v/v). Tween 80 (1%, m/v) and CVC or LNL were added to the chitosan solution, followed by Turrax homogenization at 5000 rpm for 5 min. TPP solution (0.5%, w/v) was then added rapidly to the stirred emulsion, using a syringe, and moderate agitation was continued for 10 min.

### Characterization of the chitosan nanoparticles

The hydrodynamic diameter and polydispersity index of the chitosan nanoparticles were determined by photon correlation spectroscopy. The zeta potential was measured by microelectrophoresis, using a ZetaSizer ZS90 analyzer (Malvern Instruments, UK). The measurements were performed in triplicate, at 25 °C. The nanoparticle tracking analysis (NTA) technique was used to measure the hydrodynamic diameter and concentration of the nanoparticles, using a laser with a wavelength of 532 nm and processing of the images with NanoSight v. 3.1 software. The analyses were performed in triplicate, with five measurements of 60 s. In order to ensure that different particles were analyzed in the replicates, the volume injected was greater than the capacity of the cell, hence displacing the content analyzed previously. For the DLS and zeta potential analyses, the nanoparticles were diluted 1:100 (v/v) in deionized water, while 1000-fold dilution was used for the NTA analyses.

### Determination of encapsulation efficiency

The efficiency of encapsulation of the oils in the nanoparticles was evaluated by the ultrafiltration/centrifugation method, using Millipore ultrafiltration units with exclusion pore size of 10 kDa. The concentrations of unencapsulated CVC and LNL in the filtrate were determined by HPLC, and the encapsulation efficiency was calculated as the difference between the initial concentration added (100%) and the concentration in the ultrafiltrate. The analyses (in triplicate) were performed with an UltiMate 3000 RSLCnano HPLC system (Thermo Scientific). For CVC, a Phenomenex Gemini C18 reverse phase column (100 × 4.6 mm; 2.6 μm) was used, and the mobile phase consisted of acetonitrile:water (50:50, v/v), at a flow rate of 1 mL/min. For LNL, a Phenomenex Kinetex C18 column (250 × 4.6 mm; 3 μm) was employed, and the mobile phase consisted of acetonitrile:water (65:35, v/v), at a flow rate of 1.5 mL/min. The analytical curves used for quantification could be described by the following equations:

$${\rm{CVC}}=5.4666\times -0.065\,({{\rm{r}}}^{2}=0.9989)\,{\rm{and}}\,{\rm{LNL}}=1.2262\times +1.6471\,({{\rm{r}}}^{2}=0.9990).$$

### Transmission electron microscopy

The morphology of the nanoparticles was evaluated by transmission electron microscopy, The suspension of nanoparticles was dripped onto 200 mesh copper grids and allowed to dry for 25 min. Uranyl acetate (2%) was then dripped onto the grids containing the samples, as a contrast agent. After drying, the samples were analyzed using a Zeiss LEO 906 microscope operated at 80 kV^41^.

### Biological assays using mites

The biological activity experiments were conducted in the Acarology Laboratory of UNESP/FCAV (Jaboticabal campus), using the mite species *T. urticae*, kept in greenhouse cultivations on bean plants (*P. vulgaris*). In order to avoid problems of mite resistance to pesticides, mite collection was made in areas without application of agricultural chemicals. The repellency, acaricidal, and oviposition activities of the formulations were analyzed. For evaluation of the biological activities, disks (diameter of 2.5 cm) were cut from the leaves of the host plant (*P. vulgaris*). The disks (arenas) were placed in 9 × 2 cm Petri dishes containing moistened foam (height of 1.0 cm) and a thin layer of hydrophilic cotton on the foam. In order to evaluate the repellency activity and avoid escape of the mites, the arenas were surrounded with entomological glue. The formulations were applied with a Potter spray tower calibrated at 4 lbf.in^−2^, using 2 mL of the formulation per arena, corresponding to 1.56 mg cm^−2^. Evaluation was made of the effects of the CVC and LNL formulations encapsulated in nanoparticles, as well as the unencapsulated active agents emulsified with Tween 80 (1%). After drying the products on the leaves for approximately 2 h, 10 adult female *T. urticae* mites were transferred to each arena. Each treatment was repeated 8 times and each repetition was composed of one arena, totaling 80 mites per treatment. The arenas were conditioned in a climate chamber at a temperature of 25 ± 1 °C, relative humidity of 60 ± 10%, and photoperiod of 12 h. Quantifications of dead and live mites, mites adhered to the glue barrier, and the numbers of eggs were performed 12, 24, 48, and 72 h after transfer of the mites. The mite repellency percentage (% repellency) was calculated considering the total number of mites and the number adhered to the entomological glue barrier. The acaricidal effect percentage was calculated considering the total number of mites and the number of dead mites. The effect on oviposition was calculated by dividing the total number of eggs by the number of live mites.

## Results and Discussion

### Characterization of the inclusion complexes

The kneading technique was used to obtain the inclusion complexes of β-CD with the oils, due to its effectiveness and simple implementation at the laboratory scale. Formation of the inclusion complex proceeds by removal of water molecules from the interior of the hydrophobic cavity of the cyclodextrin and their spontaneous substitution by more nonpolar molecules, which is thermodynamically favorable^19,33,42^. The complexation efficiencies for CVC and LNL were 86.2 ± 1.2% and 74.2 ± 3.5%, respectively. Both molecules were efficiently complexed by the cyclodextrin, with only small amounts of the molecules being adsorbed on the surfaces. In previous studies, higher complexation efficiencies of 91.3 and 91.7% were obtained for carvacrol and eugenol, using the freeze-drying method^33,36^. The lower efficiency achieved with the kneading method could have been due to possible volatilization losses during the maceration and drying steps^33,34^. However, the kneading method was easier and more convenient in the case of the chitosan functionalized with cyclodextrins.

#### Solubility isotherms

**Figure 1:**
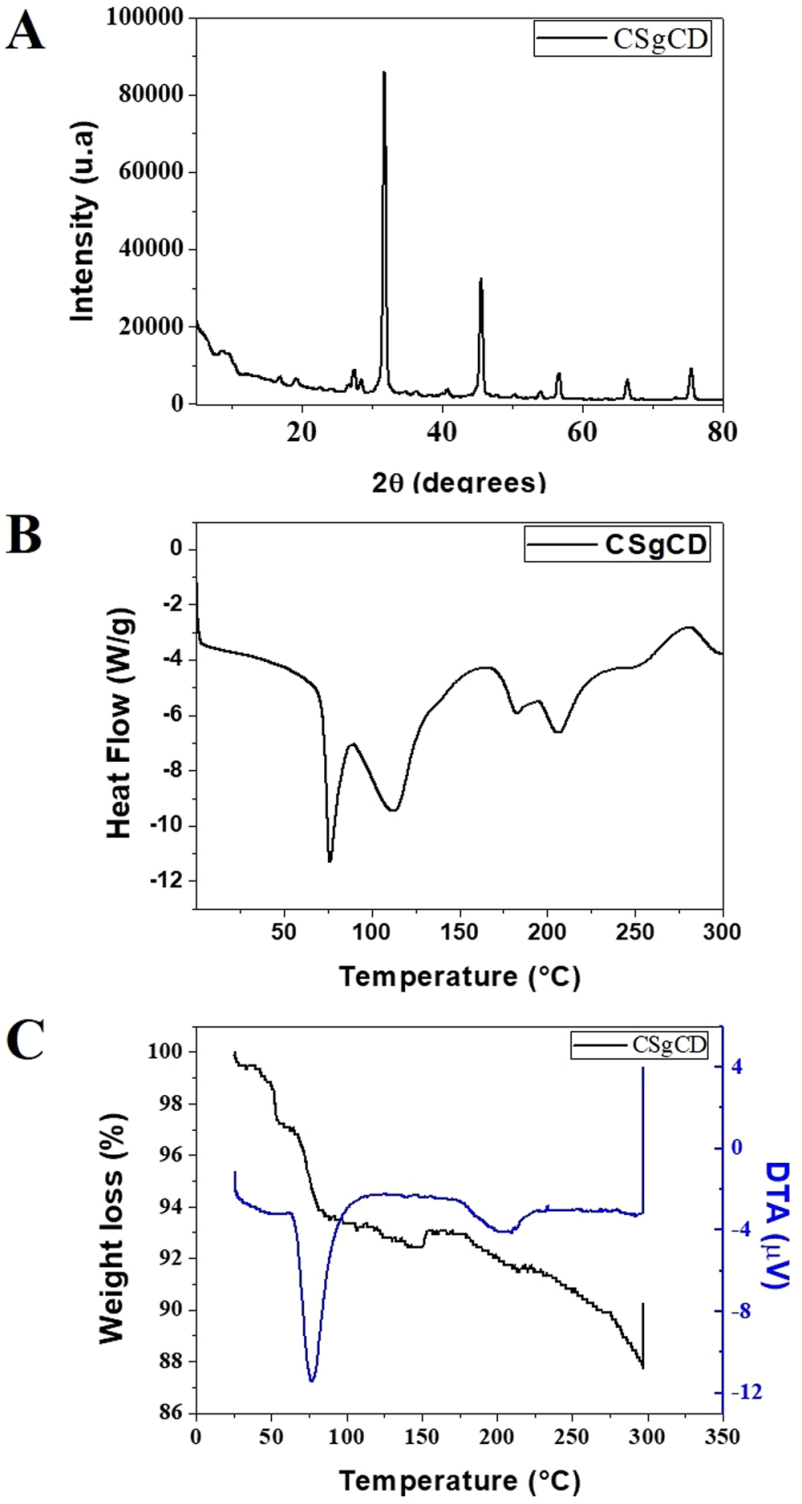
Phase solubility diagrams for CVC (**A**) and LNL (**B**) in the presence of increasing concentrations of β-CD, as a function of temperature. Experiments performed in triplicate.

The formation of inclusion complexes using cyclodextrins can provide advantages to the guest molecule including increased solubility, stabilization of the molecule in solution, and reduced losses by volatilization or photodegradation^19^. However, these effects are dependent on the stability and solubility of the complex formed. The phase solubility curves obtained for CVC (Fig. 1A) and LNL (Fig. 1B) at different temperatures (20–25 °C) were linear, with slopes less than unity, for all the temperatures tested. Such linear relationships are considered type A_L_ and here indicated that the solubilities of CVC and LNL were proportional to the increase of the cyclodextrin concentration and that the molar ratio for the formation of soluble complexes was 1:1^35^. The affinity constant of the complex (K_c_) decreased with increasing temperature, due to dissociation of the complex, since these are volatile compounds.

The values of K for carvacrol and linalool were calculated for the different temperatures, using the linear sections of the curves, with the values of the linear fits inserted in Equation 1. Table 1 shows the values of K obtained for the interactions of carvacrol or linalool with the β-CD at different temperatures.

**Table 1:** Apparent stability constant (K) values for the inclusion complexes of carvacrol and linalool with the β-CD.

K (mol.L^−1^)				
	**20 °C**	**25 °C**	**30 °C**	**35 °C**
Carvacrol	170.1 ± 0.57	157.8 ± 0.32	115.8 ± 0.86	103.4 ± 0.12
Linalool	228.2 ± 0.05	178.6 ± 1.01	152.6 ± 0.34	113.8 ± 0.54

The ΔH° and ΔS° values for the complexation of carvacrol and linalool were determined from the affinity constants for each temperature, using the van’t Hoff equation. The ΔH° and ΔS° values were −25.90 and −0.046 kJ/mol/K, respectively, for CVC, and −36.96 and −0.0806 kJ/mol/K, respectively, for LNL. The Gibbs free energies calculated from these thermodynamic parameters were −12.24 kJ/mol (carvacrol) and −12.94 kJ/mol (linalool), indicative of favorable complexation.

Similar results were reported by Santos^33^, who studied the thermodynamics of inclusion complexes of β-CD and carvacrol. It was observed that the stability constant decreased as the temperature increased, with a value of 197 M^−1^ obtained at 10 °C. The enthalpy and entropy values were both negative, indicating that the complexation of carvacrol was due to strong hydrophobic interactions and was mainly governed by enthalpic processes. Classically, the formation of inclusion complexes with cyclodextrins is associated with negative enthalpy (ΔH°) values, reflecting the existence of strong hydrophobic interactions, while entropy (ΔS°) values are negative or slightly positive, indicating that the inclusion of the guest molecule is not accompanied by strong desolvation and that formation of the complexes is governed primarily by enthalpic processes. Moraes *et al*.^43^ evaluated the influence of temperature on formation of the inclusion complex between bupivacaine and hydroxypropyl-β-cyclodextrin (HP-β-CD) and calculated the thermodynamic parameters. Different from the present findings, it was reported that the affinity constant increased as the temperature increased. The ΔH° and ΔS° values for the complexation were 4.75 and 53.48 J/mol K, respectively, and the Gibbs free energy calculated from these values was −11.19 kJ/mol. The positive values obtained for the enthalpy (ΔH°) could be explained by strong interaction between the guest molecule and the solvent, while the highly positive entropy (ΔS°) could be attributed to strong desolvation of both bupivacaine and the HP-β-CD in order to enable formation of the inclusion complex.

#### X-ray diffraction

**Figure 2:**
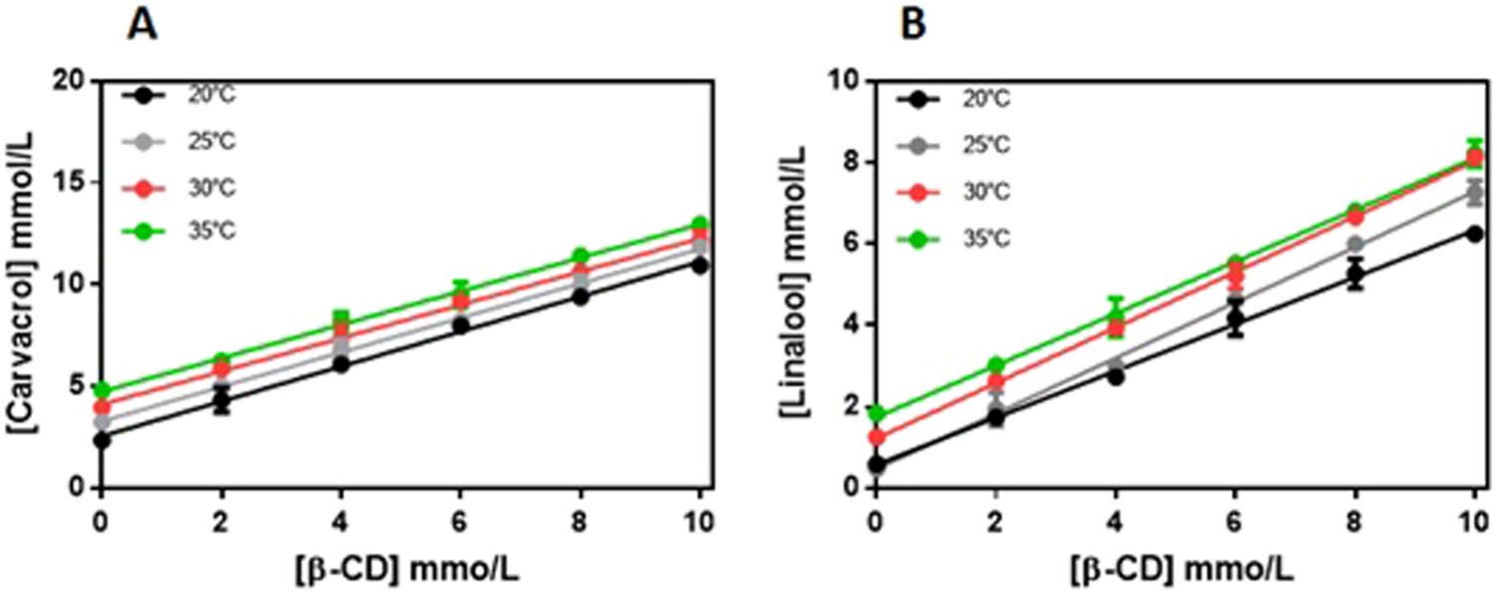
(**A**) X-ray diffractograms for the cyclodextrin (black line) and for inclusion complexes containing carvacrol (red line) and linalool (blue line). (**B**) DSC thermograms for the cyclodextrin (black line) and the inclusion complexes containing carvacrol (red line) and linalool (blue line).

The β-CD:CVC and β-CD:LNL inclusion complexes produced by the kneading method were investigated using XRD (Fig. 2A). The many narrow and intense lines present in the cyclodextrin diffractogram (Fig. 2A) were indicative of crystallinity. However, the diffractograms of the inclusion complexes (Fig. 2A) differed from the characteristic β-CD pattern. The formation of inclusion complexes can lead to changes in the diffraction patterns of the cyclodextrin and the guest molecule, such as amorphization (reduced crystallinity), disappearance of characteristic cyclodextrin peaks, and the appearance of new peaks. These features provide evidence for the formation of inclusion complexes^19,42^.

Abarca *et al*.^44^ prepared and characterized β-CD:2-NN (2-nonanone) inclusion complexes by the co-precipitation method. XRD analyses showed that the cyclodextrin peaks at 4.8° and 8° disappeared, while new peaks appeared at 12° and 18°, which could be attributed to the change in molecular organization of the cyclodextrin, with a shift from a cage-type to a channel-type structure, indicative of the formation of inclusion complexes.

#### Differential scanning calorimetry

The β-CD thermogram (Fig. 2B) showed a strong endothermic peak at 115 °C, associated with loss of water present in the hydrophobic cavity of the cyclodextrin. This peak was smaller in the thermograms of the inclusion complexes of carvacrol (Fig. 2B) and linalool (Fig. 2C), which could be explained by the displacement of water molecules by the molecules of carvacrol and linalool, indicating formation of the inclusion complex^36,45^. In addition, there were no endothermic peaks corresponding to the boiling points of carvacrol (238.7 °C) or linalool (198 °C) in the thermograms of the inclusion complexes.

Similar results were obtained by Aguiar *et al*.^42^ and Santos *et al*.^33^, who used the DSC technique to characterize inclusion complexes of β-CD with the essential oil of *Croton zehntneri* and carvacrol, respectively. In both cases, formation of the inclusion complex resulted in decreased intensity of the endothermic peak related to water loss, and no endothermic peaks corresponding to the essential oils were observed in the thermograms of the inclusion complexes.

#### Nuclear magnetic resonance analyses

Characterization of inclusion complexes should include determination of their stoichiometric formation affinity constants and their geometry. Use of nuclear magnetic resonance (NMR) enabled characterization of the inclusion complexes by means of the changes observed in the chemical shifts of the hydrogens of the cyclodextrin and guest molecules (CVC or LNL), following formation of the inclusion complexes. Table 2 presents the values of the chemical shifts obtained for carvacrol and linalool in the presence and absence of cyclodextrin, as well as the effects on the chemical shifts of the cyclodextrin hydrogens.

**Table 2:** Physicochemical properties of the NP nanoparticle systems.

Formulation	Size (nm)	PI	Zeta potential (mV)	[Particles/mL]	pH
NP	465.7 ± 45.4	0.423 ± 0.07	15.8 ± 0.90	1.31 × 10^11^ ± 8.08 × 10^9^	3.87 ± 0.05
NP_CVC	175.2 ± 2.97	0.367 ± 0.03	13.5 ± 1.38	1.08 × 10^11^ ± 1.44 × 10^10^	3.87 ± 0.01
NP_LNL	245.8 ± 29.8	0.269 ± 0.05	17.3 ± 1.37	4.8 × 10^10^ ± 5.53 × 10^9^	3.9 ± 0.01

**Figure 3:**
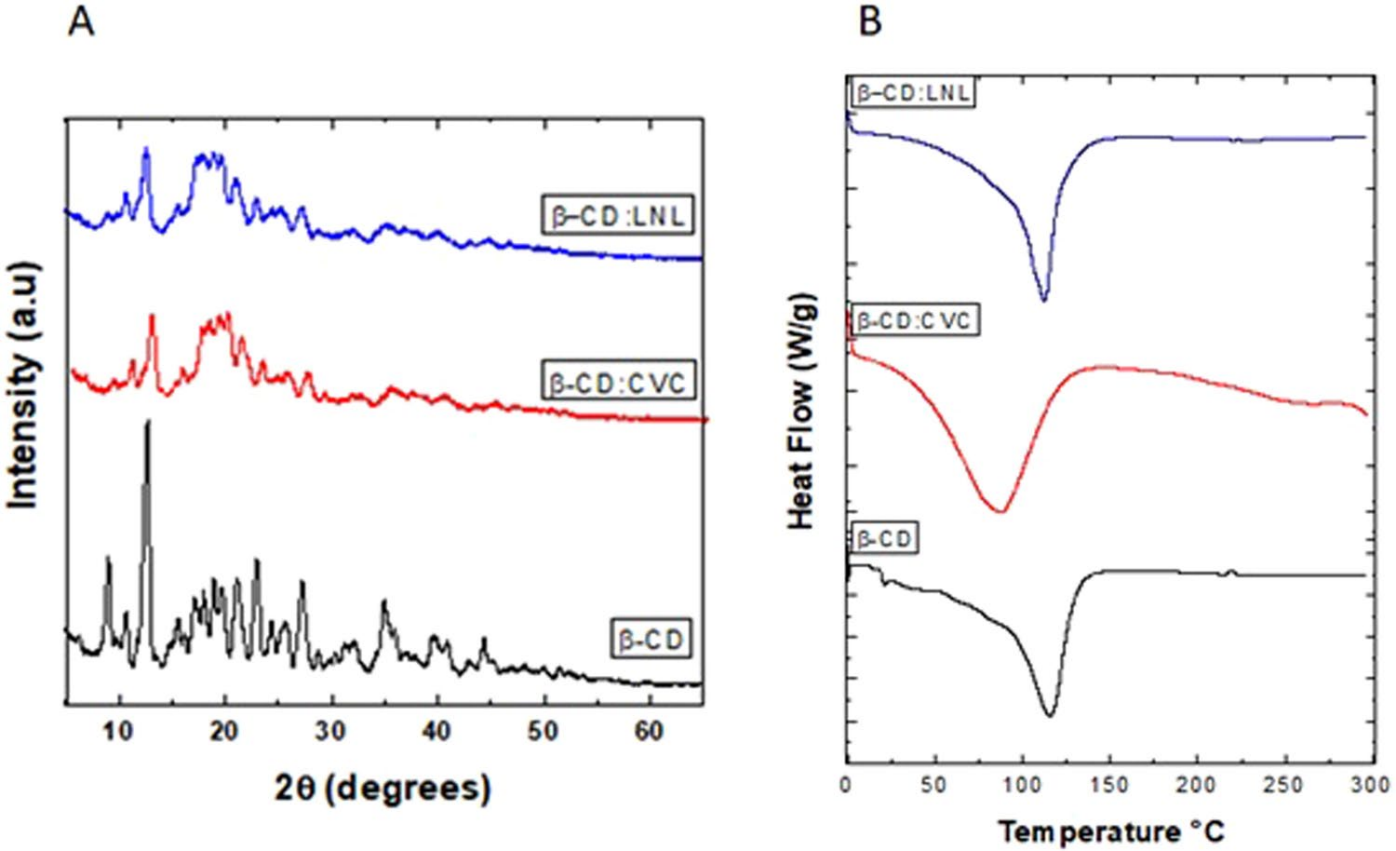
Chemical shifts (ppm) and assignment of the hydrogens of β-CD, CVC, LNL, and the CVC:β-CD (1:1) and LNL:β-CD (1:1) inclusion complexes. Values of Δδ (δ_absence_ − δ_presence_).

**Figure 4:**
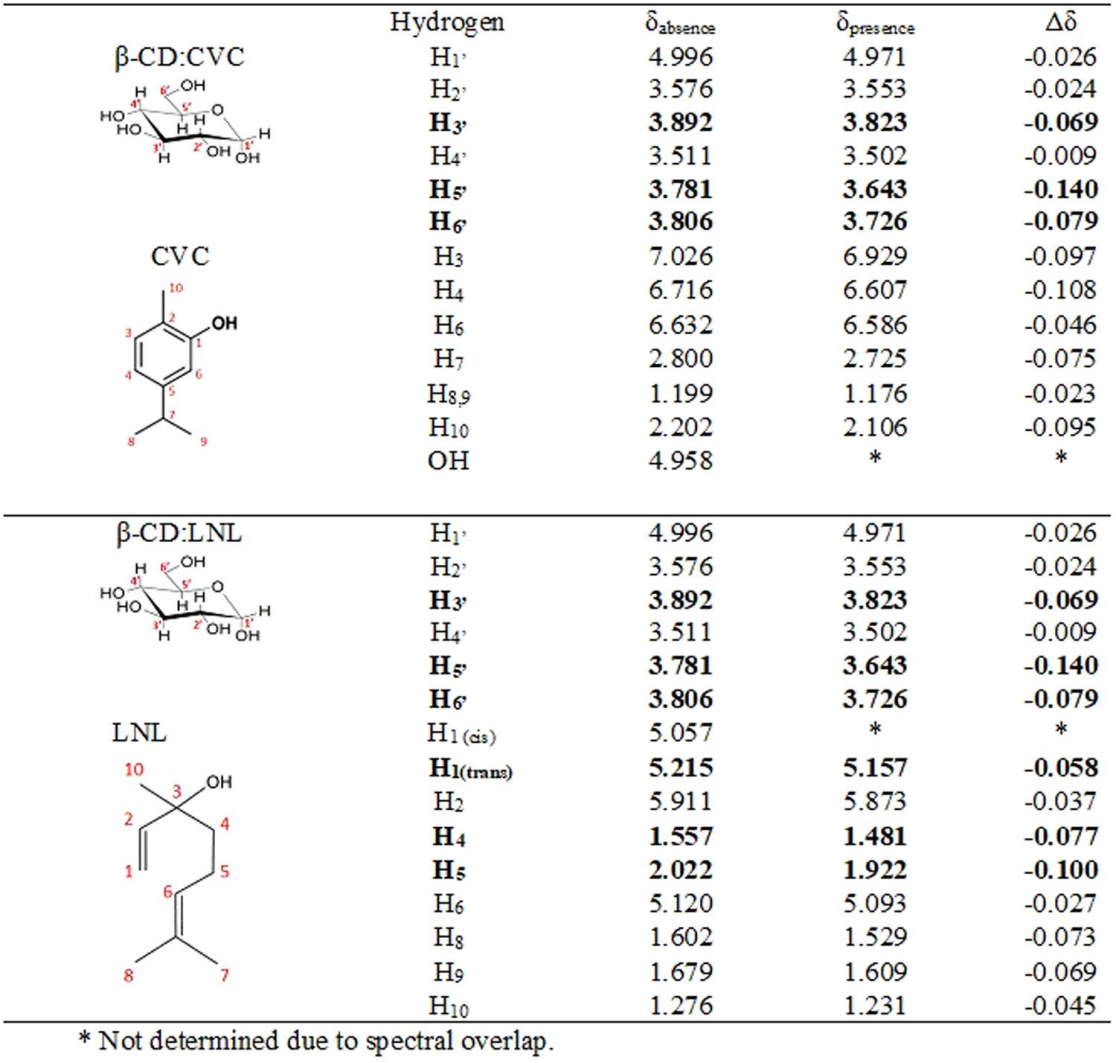
(**A**) ^1^H NMR spectra of β-CD, CVC, and the β-CD:CVC (1:1) inclusion complex. (**B**) ROESY spectra of the β-CD:CVC (1:1) inclusion complex.

Assignment of the protons of the β-CD (Figs 3 and 4A) was in agreement with the literature [18]. Changes in the chemical shifts of the cyclodextrin hydrogens were observed in the presence of CVC or LNL (Fig. 3). For both inclusion complexes, the chemical shifts of the H_3′_ and H_5′_ protons of the β-CD were greater, compared to the shifts of the other protons. Hydrogens 3 and 5 are located within the cavity of the cyclodextrin molecule and were therefore most affected by the entry of the guest molecules, indicating that both molecules interacted with the cavity^46–48^. In the case of the inclusion complex containing carvacrol, the greatest effect occurred in the lower (narrower) side of the cavity of the cyclodextrin molecule, since a large change was observed in the shift of the H_6′_ hydrogen of the β-CD. Locci *et al*.^18^ and Kfoury *et al*.^49^ also observed changes in the shift of hydrogen 6 of the cyclodextrin molecule following the formation of inclusion complexes with carvacrol.

The results demonstrated that the chemical shifts of both guest molecules were modified by formation of the inclusion complexes (Fig. 3). Changes were observed in the chemical shifts of the hydrogens associated with the aromatic rings of the carvacrol molecules, which were probably inserted within the hydrophobic cavity of the cyclodextrin. This supported the changes observed for the hydrogens in the internal cavity of the cyclodextrin molecule, since it is known that the ring current effect can induce changes in the shifts of internal hydrogens of the cyclodextrin cavity.

Similar results were obtained by Kfoury *et al*.^49^, who studied the ability of β-CD to encapsulate and solubilize carvacrol and thymol. For both molecules, it was observed that the hydrophobic interactions occurred between the hydrogen atoms present in the aromatic rings of the molecules and the hydrogens of the internal cavity of the cyclodextrin. In addition, Locci *et al*.^18^ prepared inclusion complexes of β-CD with carvacrol, eugenol, and thymol, and found that the aromatic rings of the molecules were responsible for the interactions with the host molecules.

In the case of linalool (data not shown), the hydrogens that showed the greatest chemical shift changes were H_1_, H_4_, and H_5_, which are the hydrogens located in the main chain of the molecule, indicating that these groups were probably close to the H_3_ and H_5_ hydrogens of the cyclodextrin. Numanoğlu *et al*.^50^ prepared inclusion complexes of linalool and benzyl acetate with hydroxypropyl-beta-cyclodextrin. Inclusion complex formation was confirmed by ^1^H NMR, which showed that complexation of the molecules caused changes in the chemical shifts of hydrogens 3 and 5 present in the hydrophobic cavity of the host molecule, as also found in the present work.

In order to confirm these observations, ROESY spectra were obtained in order to elucidate the presence and geometry of intermolecular interactions between β-CD and CVC or LNL. The most significant interactions identified from these spectra (Fig. 4B) were between the signals at 6.878, 6.558, and 6.539 ppm, corresponding to hydrogens H_3_, H_4_, and H_6_ of CVC, respectively, and the β-CD H_5_ signal at 3.639 ppm. In addition, the signal at 3.772 ppm, corresponding to H_3_ of the β-CD, showed interactions with the CVC H_6_ and H_9,10_ signals at 6.543 and 1.123 ppm, respectively. These results suggested that the aromatic ring of CVC was fully inserted in the hydrophobic cavity of the host molecule (S1, Supplementary Material).

Similar results were found by Locci *et al*.^18^, who used ROESY analysis to show that the isopropyl group was located near the wider extremity of the cyclodextrin cavity, while the OH group was positioned at the narrower side of the cavity.

ROESY analyses of the LNL complex (data not shown) revealed that the signal for the H_5_ hydrogen of the cyclodextrin (at 3.674 ppm) mainly interacted with the LNL signals at 5.207 ppm (H_6_) and 5.048 ppm (H_1_), while the signal for the cyclodextrin H_3_ hydrogen (at 3.802 ppm) interacted with the signal for the H_8_ hydrogen of LNL (at 1.578 ppm). There was also interaction of the H_6_ hydrogen of the CD with the -OH group (1.658 ppm) of the LNL, indicating that this group was located in the narrower side of the cyclodextrin cavity^51–53^.

### Functionalized chitosan (CSgCD)

Chitosan glycol is a commercially available chitosan derivative whose high aqueous solubility is due to the presence of the glycol group. It is widely used in biomedical applications for the sustained release of active agents and as a carrier for siRNA, among other applications. Chitosan glycol was selected for the development of the functionalized polymers because its solubility facilitated substitution of the amino group by the cyclodextrin. The polymers were functionalized according to the methodology described by Tan *et al*.^37^, and were characterized using FTIR, XRD, DSC, TG, and NMR analyses.

#### Characterization of the functionalization of chitosan glycol with cyclodextrin

Functionalization of the chitosan with cyclodextrin was characterized using nuclear magnetic resonance (NMR), X-ray diffractometry (XRD), differential scanning calorimetry (DSC), thermogravimetry (TG), and infrared spectroscopy (FTIR).

In the NMR analyses, signals for gCS between δ 4.223 and 3.450 ppm were attributed to hydrogens H_3_-H_6_ (S2A, Supplementary Material), while a singlet at δ 3.131 ppm was attributed to H_2_ and a singlet at δ 2.037 was assigned to the methyl group of gCS. These assignments were in agreement with previous results reported in the literature^54,55^. In the β-CD spectrum, the hydrogens were assigned as shown in Table 2. In the spectrum of the functionalized polymer (S2C, Supplementary Material), a doublet at δ 4.94 ppm corresponded to H_1_ of the β-CD, while a triplet at δ 3.80 ppm, a double doublet at δ 3.52 ppm, and a triplet at δ 3.47 ppm were attributed to the H_3_, H_2_, and H_4_ hydrogens of the β-CD, respectively. Peaks between δ 3.76 and 3.64 ppm were attributed to H_6_ and H_5_ of the β-CD and H_3_-H_6_ of the gCS. Additionally, a singlet at δ 2.761 ppm was attributed to hydrogens of the methyl group (CH_3_) of gCS^56,57^.

In addition to the ^1^H spectra, analyses were also made of diffusion coefficient measurements (DOSY). This technique enables evaluation of the Brownian motion of the molecules, which is essentially governed by the size of the molecule and the diffusion resistance of the medium^58,59^. Molecular diffusion coefficients have been widely used in studies of molecular interactions between macromolecules; when a molecule of lower molecular mass binds to a molecule with greater molecular mass, its translational movement decreases, resulting in a lower diffusion coefficient value^59,60^. The results showed that the diffusion rate of the β-CD molecule was 2.15 ± 0.12 × 10^−10^ m^2^/s, while association with the polymer reduced the diffusion rate to 1.80 ± 0.09 × 10^−10^ m^2^/s, indicating that the mobility of the cyclodextrin molecules decreased after functionalization with the chitosan glycol.

**Figure 5:**
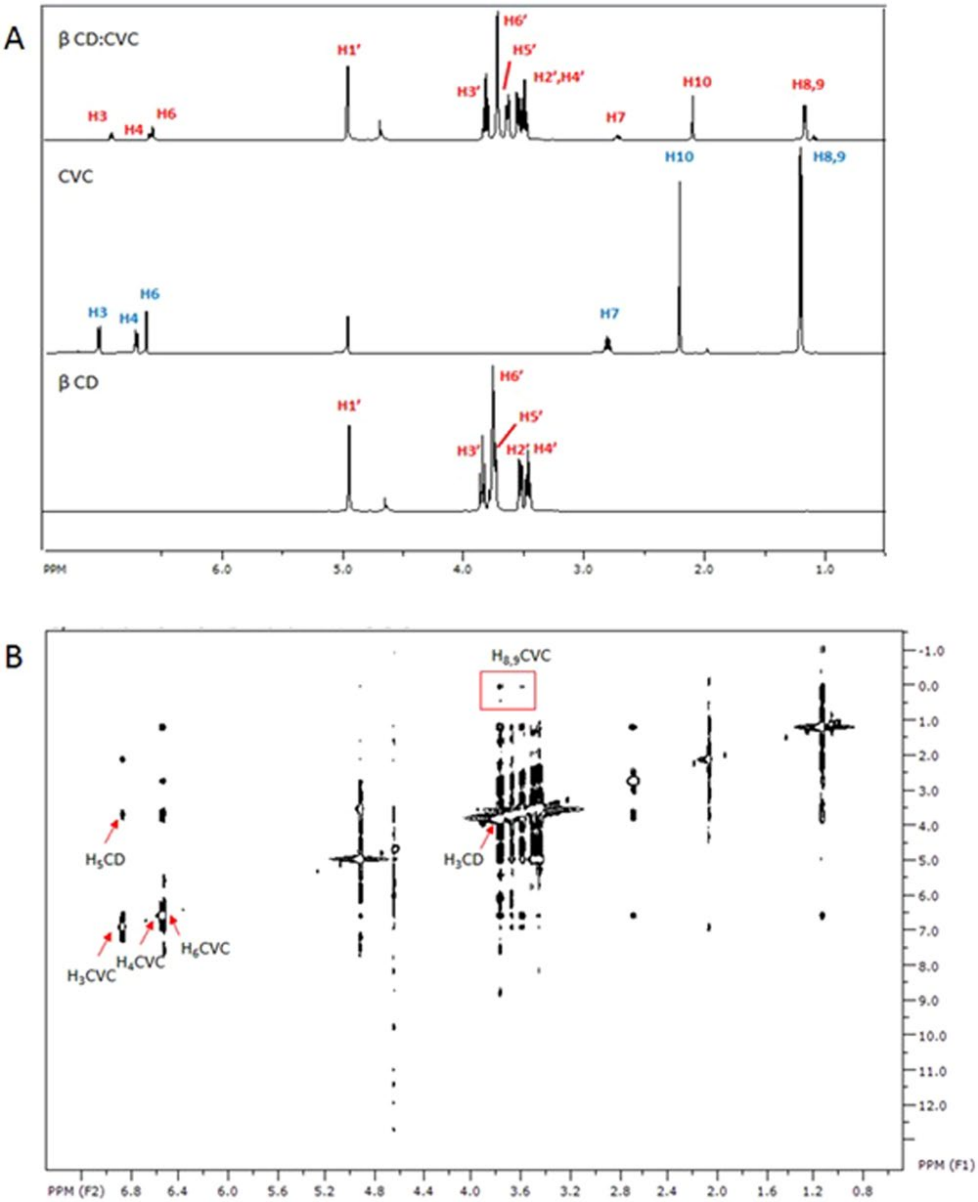
X-ray diffractogram (**A**), DSC thermogram (**B**), and TG/DTA curves (**C**) of the functionalized chitosan (CSgCD).

X-ray diffraction analysis of chitosan glycol (S3, Supplementary Material) revealed a broad peak at 20–25° associated with the semicrystalline structure of this polymer^61,62^. Analysis of the β-CD reflected the crystalline nature of this material (S3, Supplementary Material), as shown by narrow peaks at 9.14°, 10.8°, 12.6°, and 22.8°, in agreement with results reported previously^63^. In the case of functionalized chitosan (Fig. 5A), the diffractogram showed the disappearance of the β-CD lines at 9.14°, 10.8°, and 12.6°^54,61,62^, while the appearance of an intense line at 31.7° and low intensity lines at 45.5°, 56.5°, and 66.4° reflected new solid crystalline phases associated with the formation of a modified polymer of the same nature. Changes in the crystalline phase of chitosan after functionalization with cyclodextrins have also been observed elsewhere^28,32,56^.

Thermal analysis of the unmodified chitosan glycol (S4, Supplementary Material) revealed a broad endothermic peak at 92 °C, associated with the loss of hydration water of the solid state polymer, together with an exothermic peak at 265 °C, due to decomposition of the chitosan carbon chain. The cyclodextrin (S4, Supplementary Material) showed dehydration and decomposition peaks at 145 and 320 °C, respectively. The functionalized polymer (CSgCD) (Fig. 5B) showed an endothermic peak at 115 °C, associated with the evaporation of water. The shift of this peak was indicative of physical and molecular changes in the chitosan following functionalization with the cyclodextrin molecule. There was also a small increase in the thermal stability of the functionalized polymer, indicated by an increase of the temperature required for degradation of the carbon chain. Gonil *et al*.^56^ functionalized chitosan with cyclodextrin with different proportions of N-substitution (5, 11, and 23%), and DSC analyses showed that the degree of N-substitution affected the affinity of the polymer for water, as evidenced by a shift and change of area of the endothermic peak corresponding to water, compared to the unmodified chitosan.

The TG/DTA curves for chitosan glycol (S5, Supplementary Material) showed a 10% mass loss between 25 and 149 °C, corresponding to the loss of water associated with this hydrophilic polymer. The second stage of mass loss (34%), between 154 and 290 °C, corresponded to the decomposition of organic matter^64^. The β-CD (S5, Supplementary Material) showed a 15% mass loss between 27 and 95 °C, attributed to the elimination of water from the hydrophobic cavities of the cyclodextrin^48^. In the case of the functionalized polymer (Fig. 5C), the decomposition of organic matter started at a higher temperature (194 °C), compared to the non-functionalized chitosan, indicative of the effect of functionalization with the cyclodextrin. Similar results were obtained by Tan *et al*.^37^, who studied the functionalization of chitosan glycol with carboxymethyl-β-CD. Thermogravimetric data showed that the decomposition temperature of the functionalized polymer was higher than that for the chitosan but lower than for the carboxymethyl-β-CD, indicating that functionalization of the chitosan resulted in greater thermal stability.

Characterization using FTIR provided further confirmation of functionalization of the chitosan with the β-CD (S6, Supplementary Material). The β-CD spectrum presented characteristic bands including α-pyranyl vibration at 3405 cm^−1^, axial deformation of OH bonds, CH_2_ stretching at 2926 cm^−1^, stretching of C-C bonds at 1155 cm^−1^, and α-pyranyl vibration at 946.8 cm^−1^. The chitosan spectrum showed a band corresponding to β-pyranyl vibration at 888.5 cm^−1^. The spectrum of the functionalized polymer showed bands corresponding to β-CD at 945 cm^−1^ and chitosan glycol at 867.2 cm^−1^, while the band at 1744 cm^−1^ corresponding to stretching vibration of the carbonyl group almost disappeared after reaction with the amino group of the chitosan glycol. Similar results were obtained by Tan *et al*.^37^ for functionalization of chitosan glycol with carboxymethyl-β-CD.

### Chitosan/TPP nanoparticles containing LNL and CVC

The NP nanoparticles were prepared by the ionic gelation technique, in which ionic interaction between the positively charged CS and the negative charges of TPP results in the formation of inter- and intramolecular bonds. Table 2 summarizes the physicochemical characteristics of the nanoparticles prepared with the functionalized chitosan in the absence of an active agent (NP), the nanoparticles containing carvacrol (NP/CVC), and those containing linalool (NP/LNL).

Due to the low aqueous solubilities of the two active agents used in this work, the functionalized chitosan nanoparticles (NP) were produced using Tween 80 to solubilize the compounds. The mean diameter of the control nanoparticles was 465 ± 45.4 nm, while addition of the active agents led to significant reductions of the mean diameters to 175.2 ± 2.97 and 245.8 ± 29.5 nm, respectively, for the NP_CVC and NP_LNL nanoparticles. The polydispersity index values of both nanoparticulate systems containing the active agents were lower than obtained for the control nanoparticles (NP), indicating that the former nanoformulations were more homogeneous in the presence of the oils. However, it is worth noting that these were not comparable systems, since the control nanoparticles did not include any type of oil in their composition; they were formed only by the mixture of functionalized chitosan and the Tween surfactant. The zeta potential and pH values showed no significant differences between the control nanoparticles and the nanoparticles containing the active compounds.

The encapsulation efficiencies obtained for CVC and LNL were 95.3 ± 0.8% and 93.1 ± 1.2%, respectively. The higher encapsulation efficiency for CVC could be explained by the lower aqueous solubility of this compound (0.11 g/L), compared to LNL (1.589 g/L), as well as the different values of the affinity constant (170.1 ± 0.57 for CVC and 228.2 ± 0.05 for LNL) and the free energy of formation of the complex (−12.24 and −12.94 for CVC and LNL, respectively).

**Figure 6:**
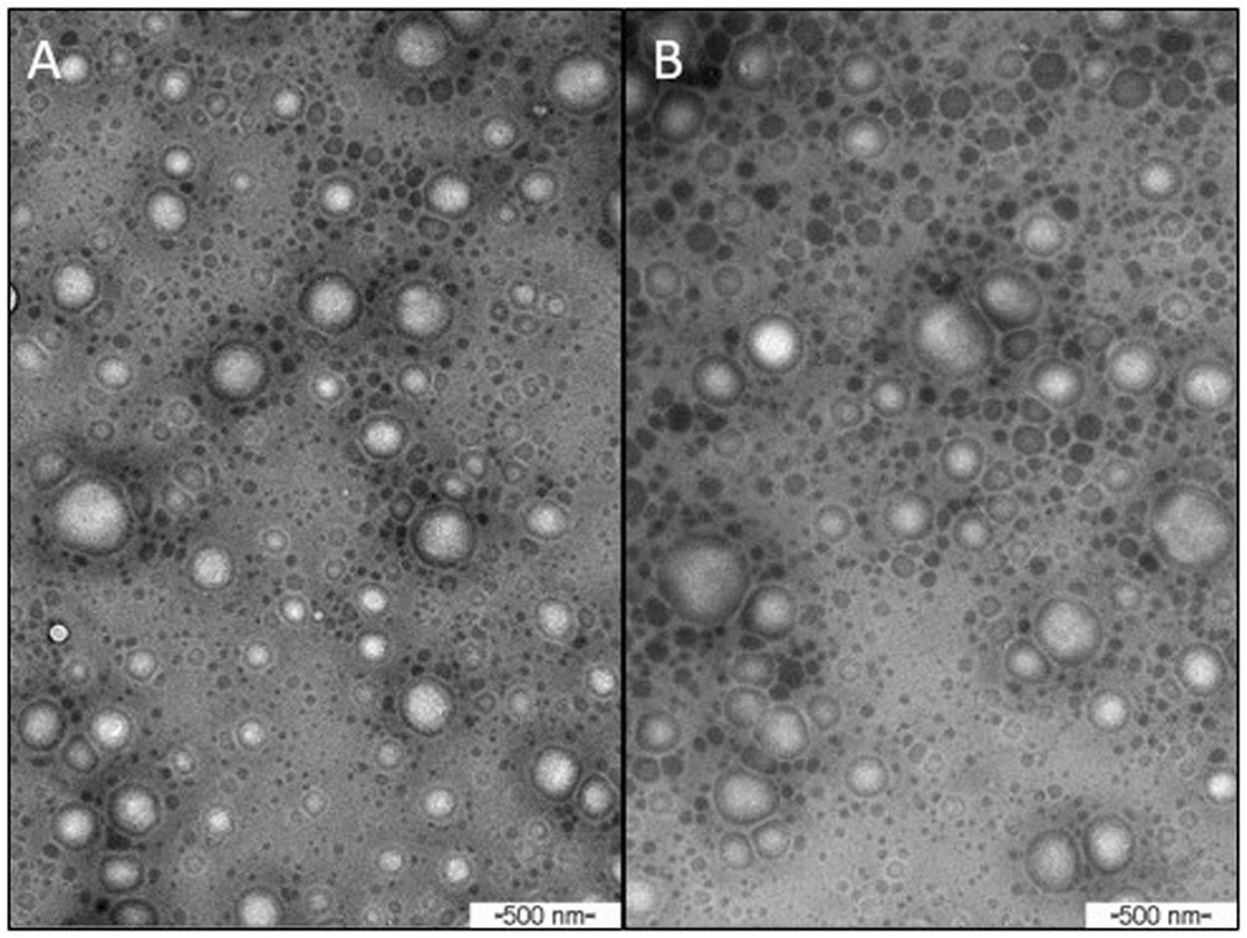
Micrographs of the chitosan nanoparticles obtained soon after preparation (initial time), at magnification of 77500×. Nanoparticles containing carvacrol (NP/CVC) (**A**) and linalool (NP/LNL) (**B**).

Morphological characterization of the nanoparticles containing the active agents by transmission electron microscopy showed that the nanoparticles were spherical (Fig. 6), with sizes of 136.3 and 175.7 nm, respectively, for the particles containing carvacrol and linalool. The diameter values were smaller than those obtained by photon correlation spectroscopy, because the TEM technique measures the diameter of dry nanoparticles, while the DLS method measures the size of the nanoparticles in solution, including the hydration barriers surrounding the nanoparticles^41^.

Yuan *et al*.^30^ prepared β-cyclodextrin-grafted chitosan nanoparticles (CDgCS) as a novel carrier for drugs with low solubility in water, using ketoprofen as a model drug. The nanoparticles were prepared by the ionic gelation method, with TPP as a crosslinking agent. The spherical nanoparticles obtained were between 202 and 589 nm in size, with polydispersity index between 0.1 and 0.3, and zeta potential between +23 and +43 mV. The encapsulation efficiency and drug loading values were in the ranges 55.4–75.3% and 13.4–16.2%, respectively. Nanoparticles with higher substitution percentage provided lower release of ketoprofen, while higher release occurred when the release medium was more acidic (pH 4.0) than when it was more basic (pH 6.8).

Izawa *et al*.^57^ investigated the carboxymethylation of α, β, and γ cyclodextrins and subsequent functionalization of chitosan. Nanoparticles were prepared containing doxorubicin crosslinked with TPP, which showed sizes of around 100 nm and the presence of aggregates (~600 nm). The efficiencies of encapsulation of doxorubicin by the nanoparticles produced with CS and the α, β, and γ cyclodextrins were 17, 17.1, 20.2, and 28.6%, respectively. The nanoparticles produced with the γ-CD-functionalized polymer formed a moderate inclusion complex with doxorubicin, resulting in an encapsulation efficiency 1.7-fold higher than obtained with CS. The higher encapsulation efficiency was attributed to ligand:host interactions as well as electrostatic interactions.

Sajomsang *et al*.^29^ prepared and characterized a mucoadhesive carrier composed of an inclusion complex between eugenol and a soluble cyclodextrin derivative grafted onto chitosan (QCD-g-CS). Different concentrations of β-CD (5–23%) were studied in order to improve solubility and mucoadhesion. The results showed that the QCD-g-CS self-aggregated at the critical aggregation concentration (CAC). The CAC values for QCD5-g-CS, QCD11-g-CS, and QCD23-g-CS were 0.156, 0.156, and 0.312 mg/mL, respectively. These self-aggregates were able to capture eugenol in both the hydrophobic β-CD cavities and the hydrophobic cores of the aggregates. Encapsulation efficiencies of 63.8, 77.5, and 59.5% were obtained for QCD5-g-CS, QCD11-g-CS, and QCD23-g-CS, respectively.

The functionalization of chitosan with cyclodextrin performed in this study resulted in materials with colloidal characteristics analogous to those described in the literature for similar systems, with higher complexation efficiency values. These systems therefore have many potential applications, for example in the biotechnological and agricultural sectors.

### Biological assays

The biological activity assays were conducted using the mite *Tetranychus urticae* Koch, which is an important agricultural pest that affects crops such as cotton, beans, and others, causing extensive damage.

The assays were performed as a function of time (0, 12, 24, 48, and 72 h), with evaluation of repellency, acaricidal activity, and effect on oviposition. Figure 6 shows the results of the assays for encapsulated CVC and LNL, as well as for CVC and LNL in emulsions with Tween 80. As discussed previously, the control nanoparticles (without the presence of oils), showed very different physico-chemical characteristics, while the presence of the oils improved the stability of the suspensions. Hence, the biological assays were not performed for these particles, in view of the differences in properties between them, which would lead to difficulties in comparing the results.

**Figure 7:**
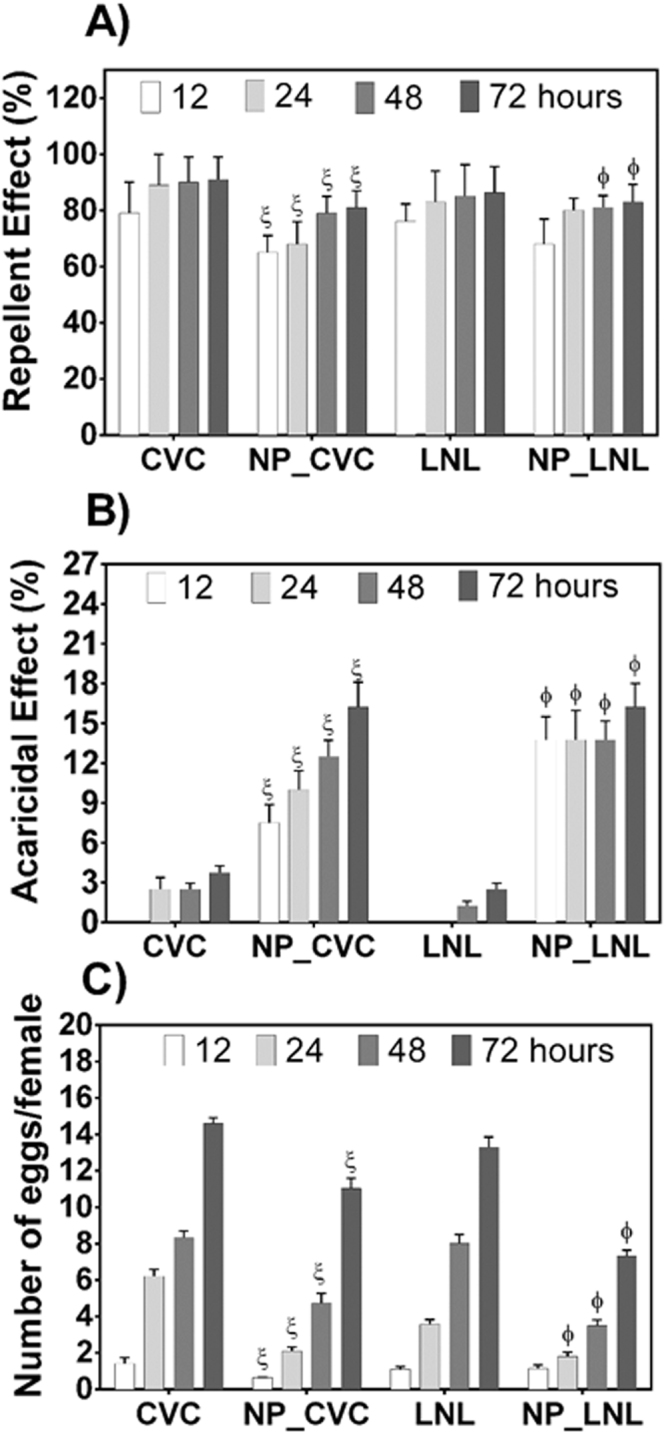
Biological effects of the botanical compounds (encapsulated or not) against the two-spotted spider mite (*Tetranychus urticae*), as a function of time. (**A**) Repellency effect, (**B**) acaricidal effect, and (**C**) effect on oviposition. A significance level of p < 0.05 was considered for the differences obtained for the same time; ^ξ^* and ^ϕ^* indicate significant differences relative to unencapsulated carvacrol and linalool, respectively.

It can be seen from Fig. 7A that the compounds presented high repellency activities against the mites, which were killed in the times studied. The nanoencapsulated compounds initially presented significantly lower effects, compared to the unencapsulated compounds, with the activities increasing as a function of time. This could have been due to modification of the release profiles of the compounds after encapsulation, since the compounds were not immediately fully available to cause an effect, but were progressively released over time^65^. However, despite their lower initial effectiveness, at the end of the trial period the encapsulated compounds showed repellency activities greater than 80%. The high repellency activities of the compounds towards *Tetranychus urticae* were in agreement with previous studies^66–71^. Pavela *et al*.^67^ studied the fumigant effect of 18 different commercial essential oils on *Tetranychus urticae*. According to the authors, the composition of the essential oils played a crucial role in the fumigant effect for all stages of development of *T. urticae*. The most effective essential oils were those of *Mentha spicata* and *Ocimum basilicum*, mainly composed of linalool and carvone (a precursor of carcavrol in the process of acid-catalyzed isomerization).

It is noteworthy that even with high repellency activity, the compounds also presented acaricidal activity and hindered oviposition (Fig. 7B,C). It should also be emphasized that when the active substances were encapsulated in the nanoparticles, there were significant increases in acaricidal activity, in addition to significant decreases in oviposition. At the final time studied (72 h), CVC and LNL presented acaricidal activities of 3.75 ± 0.51 and 2.5 ± 0.47%, respectively, whereas the encapsulated compounds presented values of 15.35 ± 1.87 and 16.25 ± 2.02%, respectively. In the case of oviposition, at 72 h the encapsulated CVC and LNL decreased the amounts of eggs per female by 1.3 ± 0.2 and 1.8 ± 0.1 times, compared to the unencapsulated CVC and LNL, respectively. The greater effectiveness of the nanoparticle formulations could have been due to improved stability of the compounds after encapsulation, in addition to the modified release, allowing the compounds to remain at concentrations causing effective toxicity towards the mites. These results were similar to those found by Ebadollahi *et al*.^65^, who evaluated the acaricidal activity (against *Tetranychus urticae*) of the essential oils of *Thymus eriocalyx* and *Thymus kotschyanus* nanoencapsulated in mesoporous material (MCM-41). According to the authors, the nanoencapsulation of the essential oils increased their effectiveness about 4-fold, compared to the unencapsulated compounds. It was also suggested that the observed effects were due to decreased degradation of the compounds and the slow release of the active substances in the essential oils.

Ziaee *et al*.^72^ encapsulated the essential oil of *Carum copticum* in nanogels of chitosan/myristic acid and evaluated its effects on two agricultural pests, *Sitophilus granarius* and *Tribolium confusum*. According to the findings, the oil-loaded nanogels were 8.9 and 3.7 times more toxic than the unencapsulated oil against *S. granarius* and *T. confusum*, respectively. The nanoencapsulation also increased the effectiveness of the oil as a function of time. These results were ascribed to increased oil stability (decreased degradation), as well as sustained release of the compound as a function of time.

In summary, both CVC and LNL, whether nanoencapsulated or not, showed high repellency against *Tetranychus urticae*. However, use of the encapsulated compounds resulted in significant increases in acaricidal activity, as well as decreases in oviposition. It should be noted that all the formulations presented significant differences, relative to the control (Table S1, Supplementary Information). Hence, the prepared nanoparticles containing these essential oils were shown to be promising for the control of the mite, since the formulations simultaneously presented repellency and acaricidal effects. Tak *et al*.^66^ studied the acaricidal and repellency activities of active compounds derived from plant essential oils towards *Tetranychus urticae* Koch, and also observed simultaneous acaricidal and repellency effects of carvacrol and linalool. However, it was suggested that a number of factors could greatly affect the toxicity or repellency of the active compounds. These included the effects of vapor pressure, interaction with treated surfaces (speed/degree of metabolism and penetration into leaf structures), test methods (direct contact, residual contact, or spraying), and the interaction between the compounds and the target species (including the effects of body size, degree of penetration, and delivery).

## Conclusions

Beta-cyclodextrin inclusion complexes were produced with carvacrol (a botanical insecticide) and linalool (a botanical repellent) as guest molecules. Characterization of the materials by different methods confirmed formation of the inclusion complexes. The β-CD complexation efficiency obtained for carvacrol was higher than for linalool, although high efficiencies were observed in both cases. Nanoparticulate systems based on natural polysaccharides (chitosan and cyclodextrin) were prepared using the ionic gelation method and were used as carriers for carvacrol and linalool. This is the first report concerning the use of nanoparticulate systems of chitosan functionalized with cyclodextrins to carry these low aqueous solubility compounds. The nanoformulations presented good colloidal characteristics (size, polydispersity, and zeta potential) and high encapsulation efficiencies for both active agents. The findings suggest that these nanocarrier systems may be good candidates for improving the lifetimes of carvacrol and linalool when exposed to the environment. Nanoencapsulated carvacrol and linalool showed repellency, acaricidal, and oviposition activities against *Tetranychus urticae*. The results open perspectives for the encapsulation of other active agents, since hydrophilic substances can be transported in the polymeric chitosan matrix, while hydrophobic substances can be carried in the cavities of the cyclodextrins present on functionalized chitosan, offering the potential for applications in different areas, including agriculture.

## Electronic supplementary material

Supplementary Material
