# Publisher Correction: Chitosan nanoparticles functionalized with β-cyclodextrin: a promising carrier for botanical pesticides

**DOI:** 10.1038/s41598-018-25618-y

**Published:** 2018-05-16

**Authors:** Estefânia V. R. Campos, Patrícia L. F. Proença, Jhones L. Oliveira, Cirano C. Melville, Jaqueline F. Della Vechia, Daniel J. de Andrade, Leonardo F. Fraceto

**Affiliations:** 1 Department of Environmental Engineering, São Paulo State University (UNESP), Sorocaba, SP Brazil; 2 Department of Biochemistry and Tissue Biology, State University of Campinas, Campinas, SP Brazil; 3 São Paulo State University (UNESP), College of Agricultural and Veterinary Sciences, Jaboticabal, SP Brazil

Correction to: *Scientific Reports* 10.1038/s41598-018-20602-y, published online 01 February 2018

This Article contains an error in the order of the Figures. Figures 1, 2, 3, 4 and 5 were published as Figures 2, 3, 4, 5 and 1 respectively. The correct Figures 1, 2, 3, 4 and 5 appear below. The Figure legends are correct.

**Figure 1:**
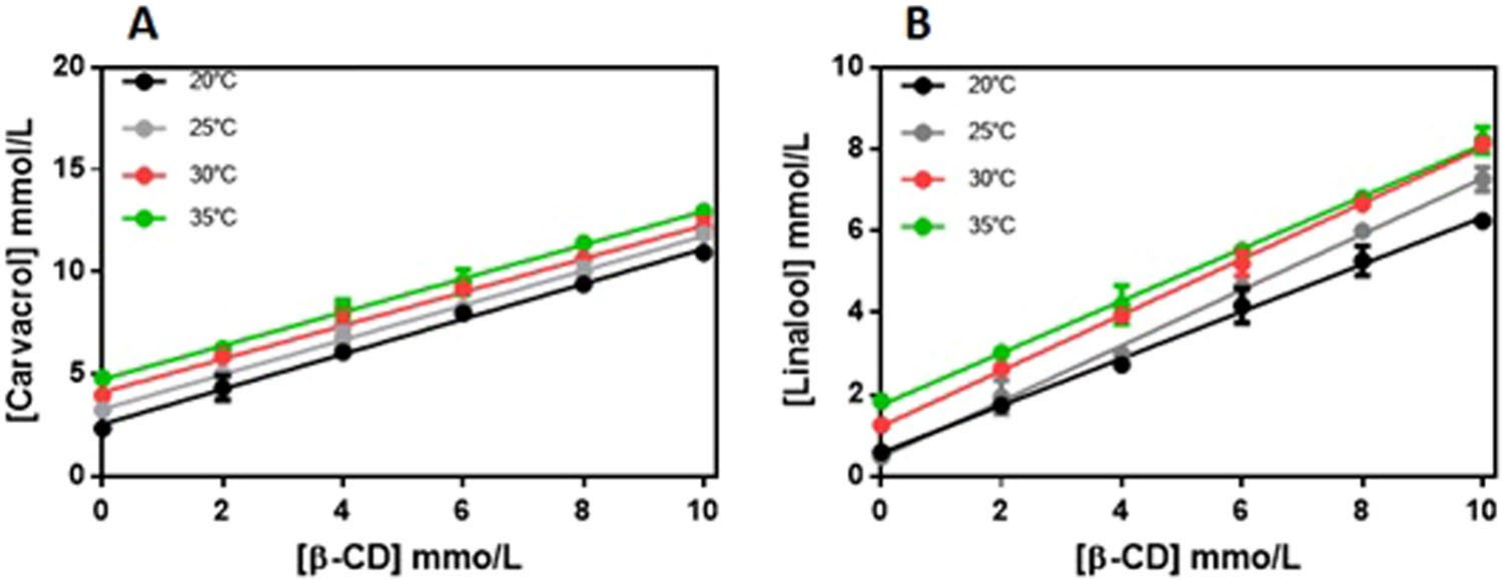
Phase solubility diagrams for CVC (**A**) and LNL (**B**) in the presence of increasing concentrations of β-CD, as a function of temperature. Experiments performed in triplicate.

**Figure 2:**
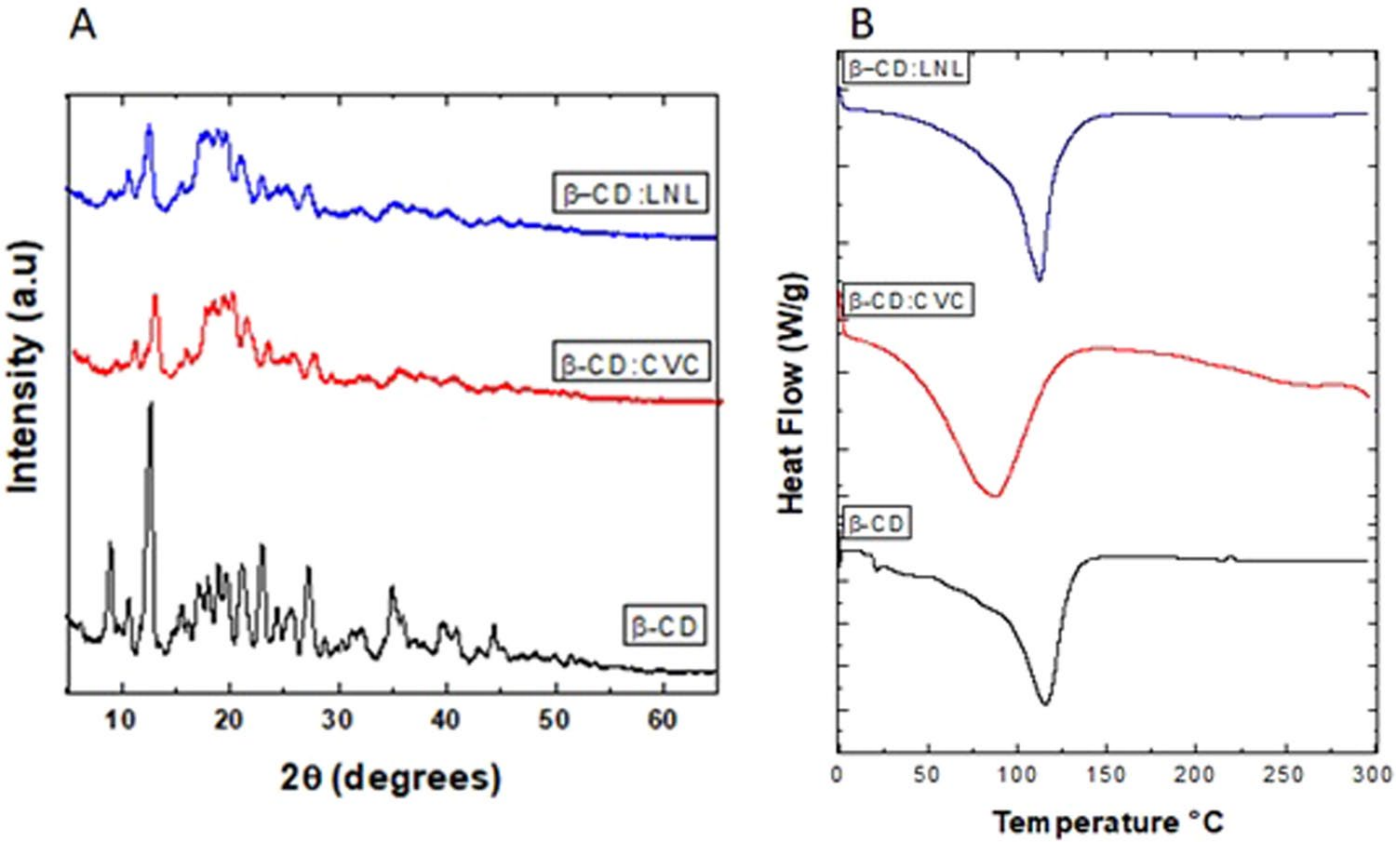
(**A**) X-ray diffractograms for the cyclodextrin (black line) and for inclusion complexes containing carvacrol (red line) and linalool (blue line). (**B**) DSC thermograms for the cyclodextrin (black line) and the inclusion complexes containing carvacrol (red line) and linalool (blue line).

**Figure 3:**
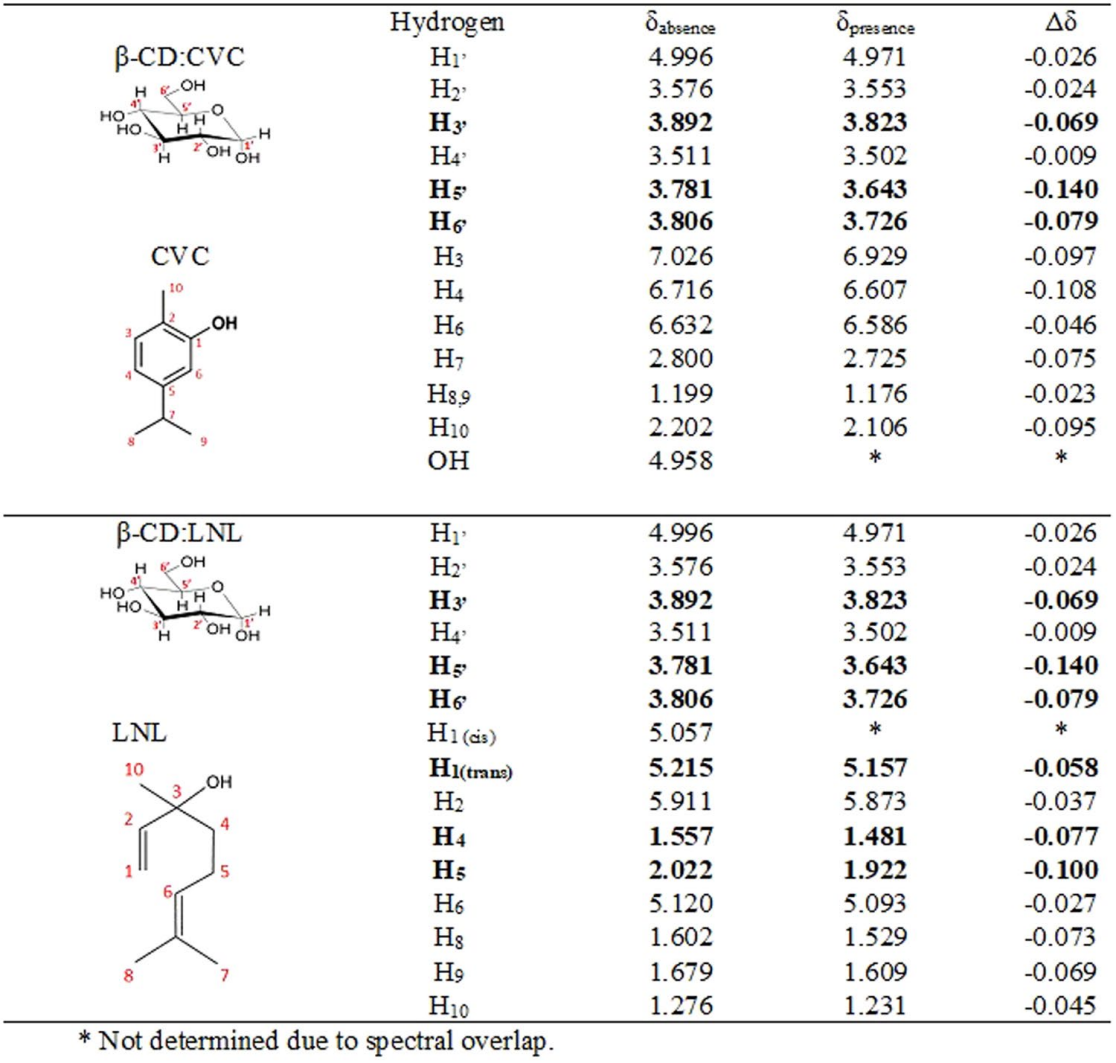
Chemical shifts (ppm) and assignment of the hydrogens of β-CD, CVC, LNL, and the CVC:β-CD (1:1) and LNL:β-CD (1:1) inclusion complexes. Values of Δδ (δ_absence_ − δ_presence_).

**Figure 4:**
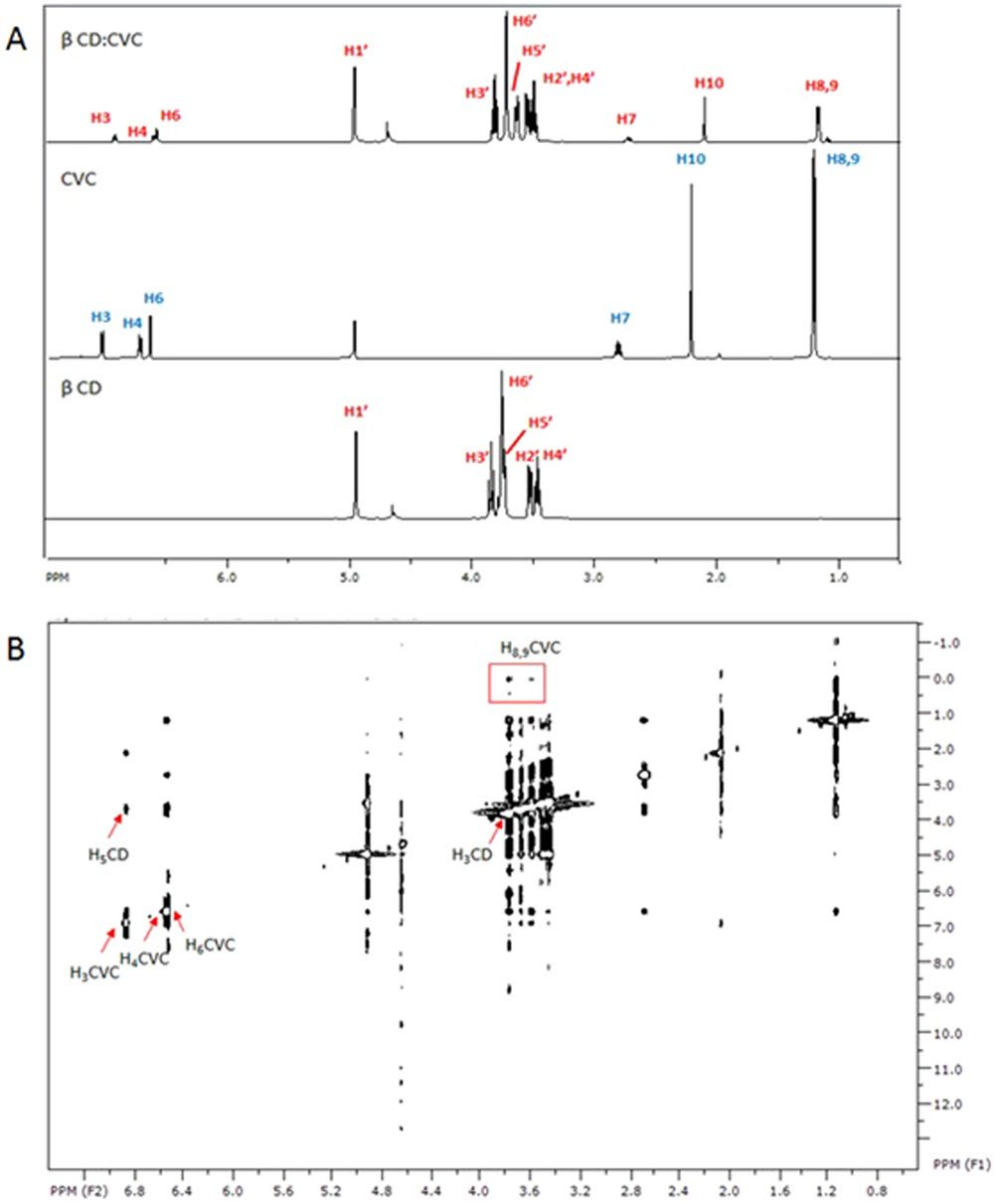
(**A**) ^1^H NMR spectra of β-CD, CVC, and the β-CD:CVC (1:1) inclusion complex. (**B**) ROESY spectra of the β-CD:CVC (1:1) inclusion complex.

**Figure 5:**
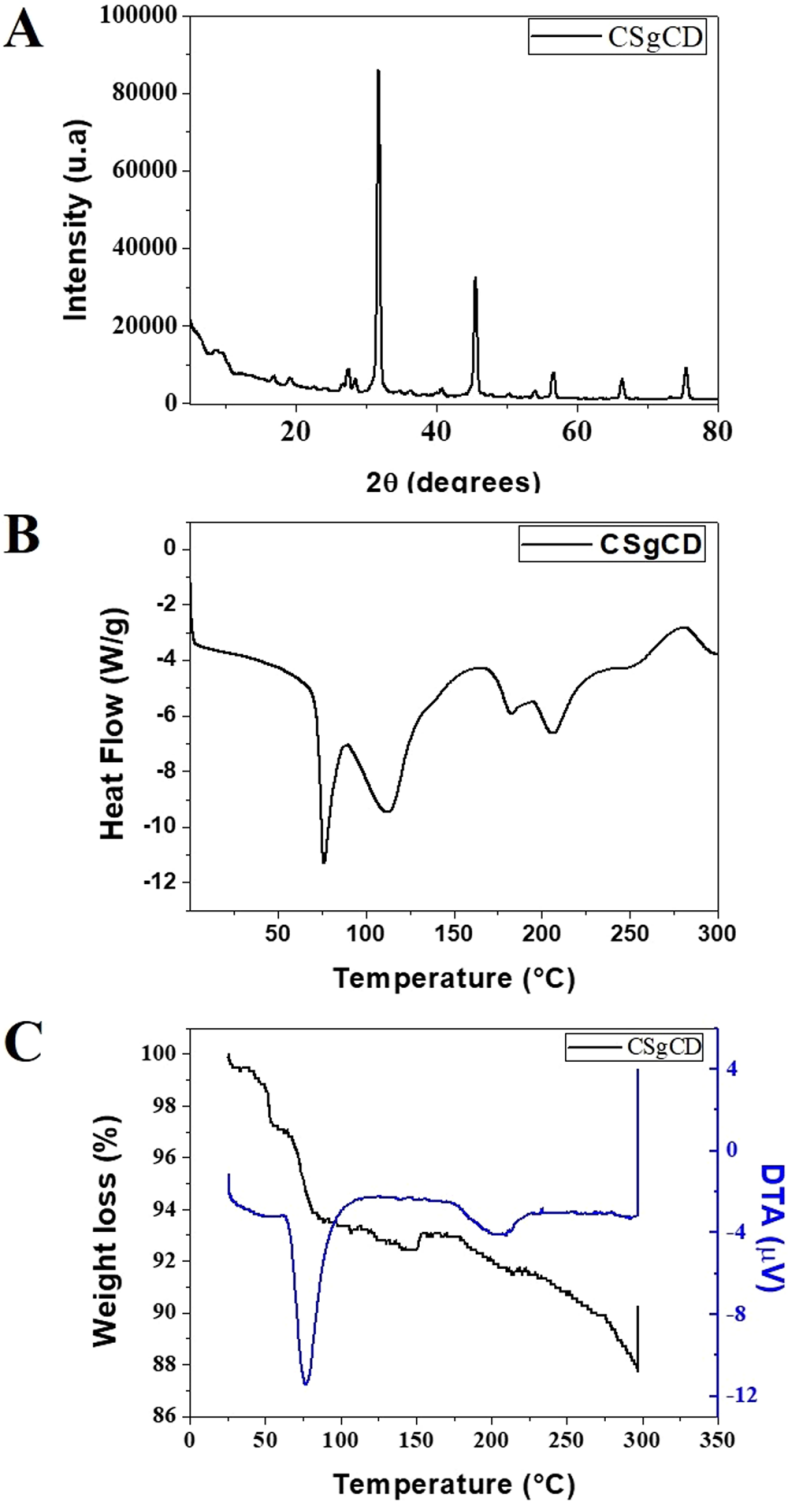
X-ray diffractogram (**A**), DSC thermogram (**B**), and TG/DTA curves (**C**) of the functionalized chitosan (CSgCD).

