# Supplementary material for: Chitosan nanoparticles functionalized with β-cyclodextrin: a promising carrier for botanical pesticides
Source: Sci Rep. 2018 Feb 1;8:2067. doi: 10.1038/s41598-018-20602-y (PMC5794797; doi:10.1038/s41598-018-20602-y)
Supplement: Supplementary file 1 — Supplementary Material [file 41598_2018_20602_MOESM1_ESM.pdf]

SUPPLEMENTARY MATERIAL

Chitosan nanoparticles functionalized with β-cyclodextrin: a promising carrier for botanical pesticides

Estefânia V. R. Campos[1,2], Patrícia L. F. Proença[1], Jhones L. Oliveira[1], Cirano C. Melville[3], Jaqueline F. Della Vechia[3], Daniel J. de Andrade[3], Leonardo F. Fraceto[1,2] *

[1]Department of Environmental Engineering, São Paulo State University (UNESP), Sorocaba, SP, Brazil

[2]Department of Biochemistry and Tissue Biology, State University of Campinas, Campinas, SP, Brazil

[3]São Paulo State University (UNESP), College of Agricultural and Veterinary Sciences, Jaboticabal, SP, Brazil

[*]Corresponding author: São Paulo State University, Av. Três de Março, 511, Alto da Boa Vista, CEP 18087-180, Sorocaba, São Paulo, Brazil. E-mail: leonardo@sorocaba.unesp.br

S1: Schematic representations of the geometries of the inclusion complexes of the β-cyclodextrin with (A) CVC and (B) LNL.

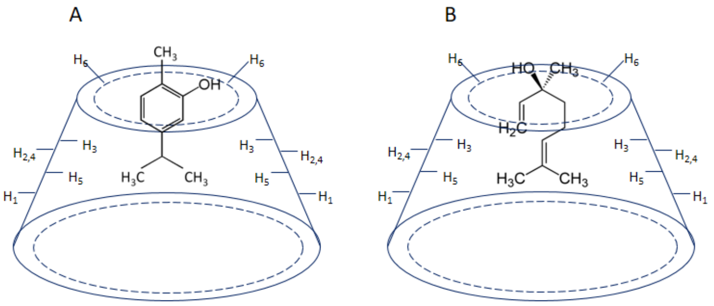

S2: ^{1}H NMR spectra of chitosan glycol (gCS), the beta-cyclodextrin (β-CD), and functionalized chitosan (CSgCD), at 25 ^{o}C and 500 MHz.

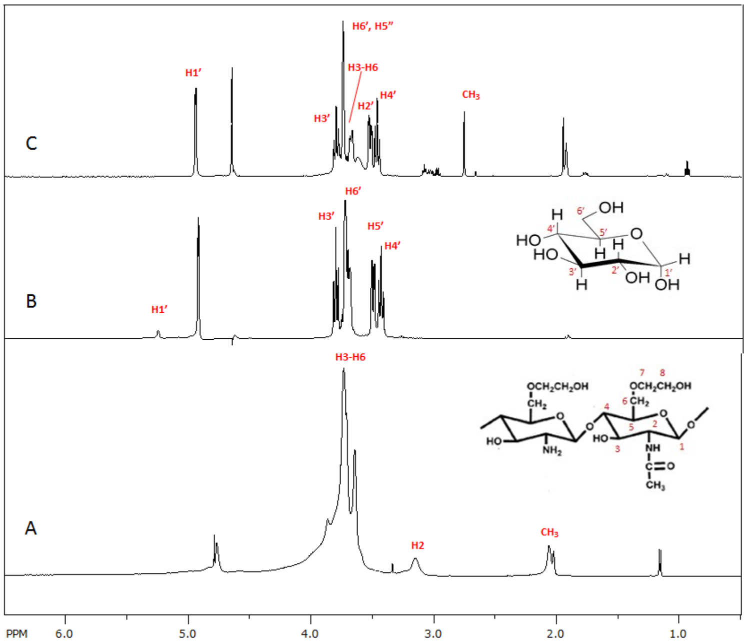

S3: X-ray diffractograms of chitosan glycol (A) and the beta-cyclodextrin (B).

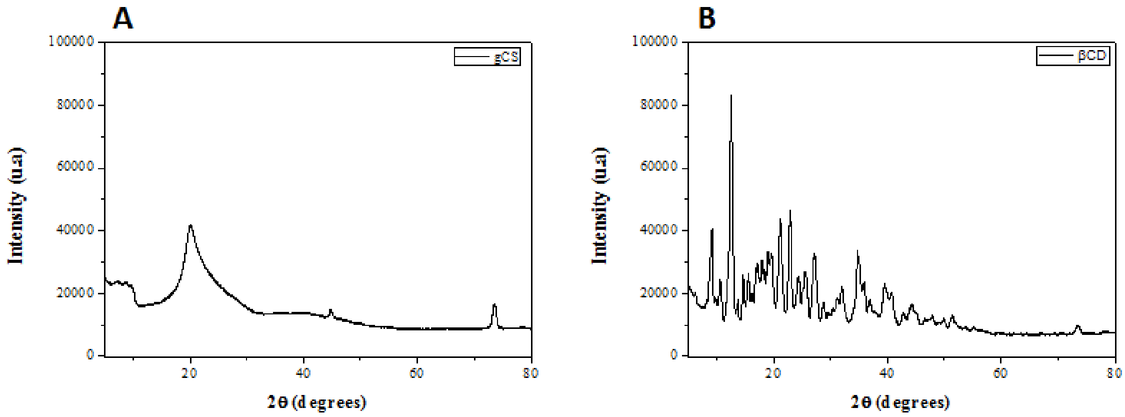

S4: DSC thermograms of chitosan glycol (A) and the beta-cyclodextrin (B).

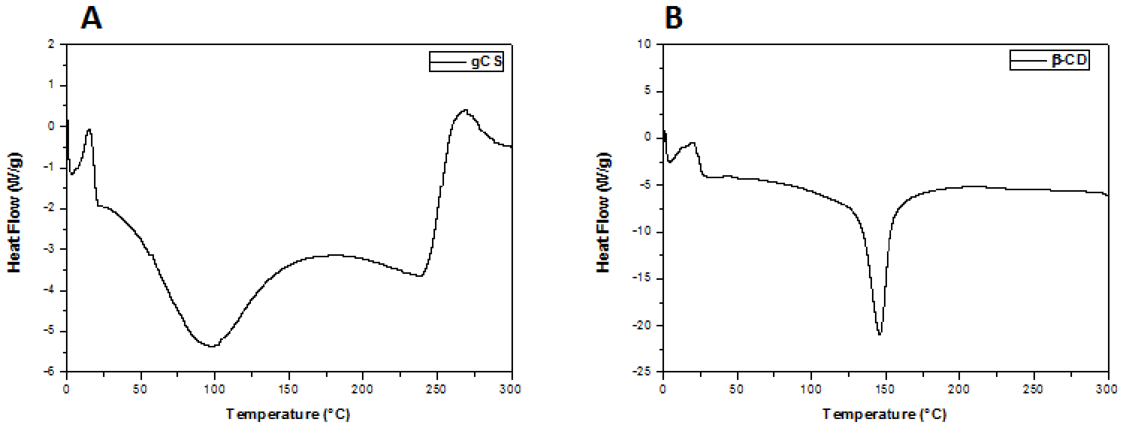

S5: TG/DTA curves of chitosan glycol (A) and the beta-cyclodextrin (B).

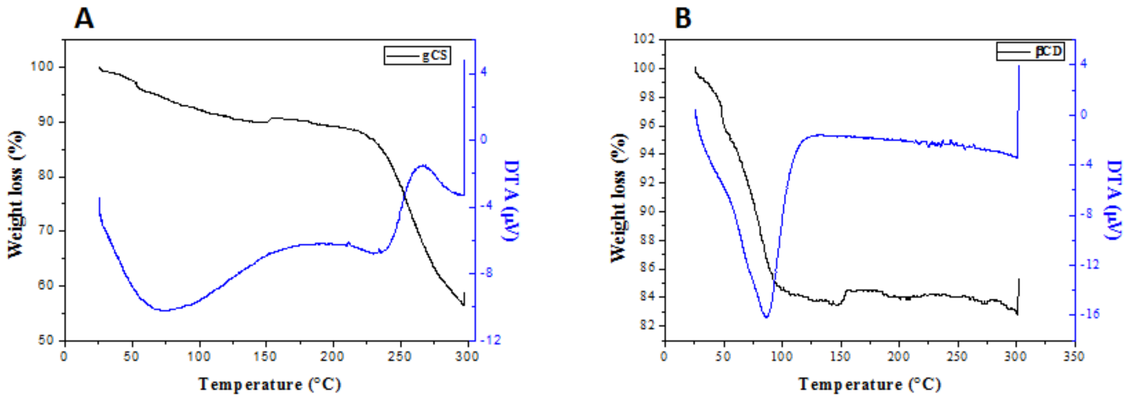

S6: Infrared spectra for the β-CD (A), chitosan glycol (B), and the functionalized polymer (C).

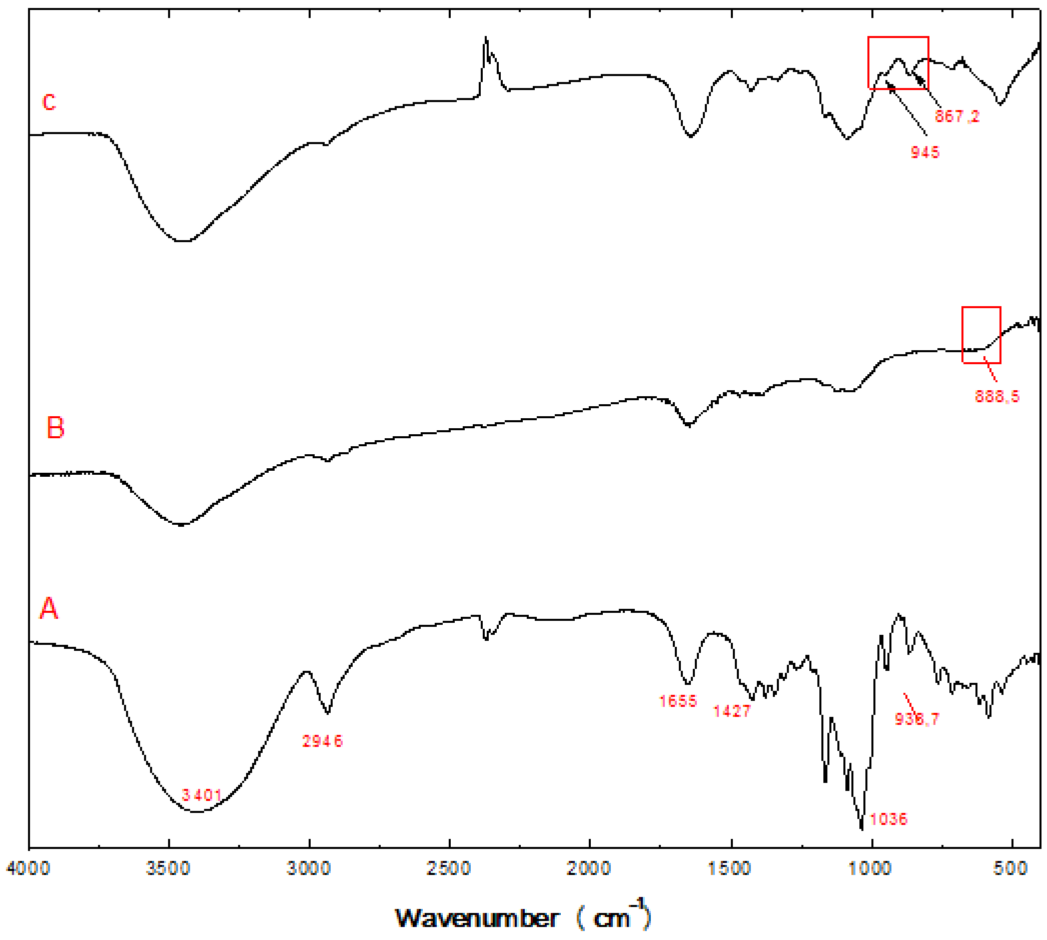

Table S1: **Table S1:** Biological effects of the control (water) against two-spotted spider mite (*Tetranychus urticae*) as function of time.

	Time (hours)	Biological Effects		
		**Repellent Effect (%)**	**Acaridical Effect (%)**	**Effect on oviposition (eggs/female)**
**Control**	12	0	0	3,47 ± 1,1
	24	4,2 ± 1,2	0	5,5 ± 2,1
	48	4,1 ± 2,2	0	12,9 ± 2,6
	72	11,3 ± 3,5	0	22,5 ±3,1
